# A persistently replicating SARS-CoV-2 variant derived from an asymptomatic individual

**DOI:** 10.1186/s12967-020-02535-1

**Published:** 2020-09-23

**Authors:** Francesca Caccuri, Alberto Zani, Serena Messali, Marta Giovanetti, Antonella Bugatti, Giovanni Campisi, Federica Filippini, Erika Scaltriti, Massimo Ciccozzi, Simona Fiorentini, Arnaldo Caruso

**Affiliations:** 1 Department of Molecular and Translational Medicine, Section of Microbiology, University of Brescia, Brescia, Italy; 2 Flavivirus Laboratory, Oswaldo Cruz Institute, Oswaldo Cruz Foundation, Rio De Janeiro, Brazil; 3 Unit of Medical Statistics and Molecular Epidemiology, University Campus Bio-Medico of Rome, Rome, Italy

**Keywords:** Virus persistence, SARS-CoV-2, Asymptomatic infection, Virus isolate, Genetic variation, COVID-19 epidemic

## Abstract

**Background:**

Since the first outbreak of SARS-CoV-2, the clinical characteristics of the Coronavirus Disease 2019 (COVID-19) have been progressively changed. Data reporting a viral intra-host and inter-host evolution favouring the appearance of mild SARS-CoV-2 strains are since being accumulating. To better understand the evolution of SARS-CoV-2 pathogenicity and its adaptation to the host, it is therefore crucial to investigate the genetic and phenotypic characteristics of SARS-CoV-2 strains circulating lately in the epidemic.

**Methods:**

Nasopharyngeal swabs have been analyzed for viral load in the early (March 2020) and late (May 2020) phases of epidemic in Brescia, Italy. Isolation of SARS-CoV-2 from 2 high viral load specimens identified on March 9 (AP66) and on May 8 (GZ69) was performed on Vero E6 cells. Amount of virus released was assessed by quantitative PCR. Genotypic characterization of AP66 and GZ69 was performed by next generation sequencing followed by an in-depth in silico analysis of nucleotide mutations.

**Results:**

The SARS-CoV-2 GZ69 strain, isolated in May from an asymptomatic healthcare worker, showed an unprecedented capability of replication in Vero E6 cells in the absence of any evident cytopathic effect. Vero E6 subculturing, up to passage 4, showed that SARS-CoV-2 GZ69 infection was as productive as the one sustained by the cytopathic strain AP66. Whole genome sequencing of the persistently replicating SARS-CoV-2 GZ69 has shown that this strain differs from the early AP66 variant in 9 nucleotide positions (C2939T; C3828T; G21784T; T21846C; T24631C; G28881A; G28882A; G28883C; G29810T) which lead to 6 non-synonymous substitutions spanning on ORF1ab (P892S; S1188L), S (K74N; I95T) and N (R203K, G204R) proteins.

**Conclusions:**

Identification of the peculiar SARS-CoV-2 GZ69 strain in the late Italian epidemic highlights the need to better characterize viral variants circulating among asymptomatic or paucisymptomatic individuals. The current approach could unravel the ways for future studies aimed at analyzing the selection process which favours viral mutations in the human host.

## Background

Severe acute respiratory syndrome coronavirus 2 (SARS-CoV-2) is the first pandemic coronavirus in the history coming to our observation [1]. Since the outbreak of SARS-CoV-2 in Wuhan, China, the clinical characteristics of the Coronavirus Disease 2019 (COVID-19) became progressively different, gradually evolving from clinically evident pulmonary or flu-like symptoms to subclinical or even asymptomatic infections [2–5]. Different clinical outcomes in COVID-19 patients were also described over the time at different locations around the globe. In Italy, the clinical picture of SARS-CoV-2 infection considerably changed in May 2020 as compared to the beginning of SARS-CoV-2 epidemic at the end of February 2020, with a significant reduction in the number of new cases paralleled by a decrease in the number of severe cases needing ventilatory support in intensive care units [6]. Of interest, a milder clinical picture was also seen in the oldest age cohort, experiencing worse clinical outcomes during the first phase of the Italian outbreak [7].

Genetic drift almost inevitably leads, during time, to amino acid mutations in proteins critical for virus replication and spreading, mostly generating an attenuated viral progeny [8]. Indeed, different epidemiological and clinical features of COVID-19 were found to be related to genetic changes of SARS-CoV-2 [9]. The novel coronavirus has been found to evolve into two subtypes, L and S [10], with the former being more aggressive and spreading more rapidly than the latter [9]. Jin et al. [3] compared the complete genome of 52 strains of SARS-CoV-2 and described a continuous evolution of the potential furin cleavage site of the S protein of SARS-CoV-2 till the latest isolate (ZJ01) derived from a patient with mild COVID-19. Further informatic analyses highlighted the relative high number of mutations of ZJ01 compared to the sequences of other strains of SARS-CoV-2 collected at the early stage of China epidemic. Mutations observed in ZJ01 had a direct negative impact on viral load and cytopathicity when infecting Vero E6 cells, leading the authors to hypothesize a potential change in evolutionary direction possibly promoting the appearance of a mild SARS-CoV-2 subtype. More recently, Yao et al. [11] reported the functional characterization of 11 patient-derived viral isolates showing significant variation in cytopathic effects and viral load, suggesting that patient-derived mutations have an impact on SARS-CoV-2 pathogenicity. Interestingly, plaque purification of SARS-CoV-2 cultured in Vero E6 showed that a virus isolate contains a series of quasispecies which differ in their in vitro cytopathic activity and in vivo aggressiveness, with attenuated variants being characterized by deletions at the S1/S2 junction [12]. Therefore, attenuated SARS-CoV-2 variants are already being circulating, at least as subdominant strains, in infected individuals. For this reason, investigating the pattern and frequency of mutations occurred in SARS-CoV-2 in more recently infected patients and in asymptomatic individuals is urgently needed.

## Methods

### Detection of SARS-CoV-2

Nasopharyngeal specimens were collected from the end of February to the end of May 2020 at the Brescia Civic Hospital, (Brescia, Lombardy, Italy), using FLOQSwabs in the universal transport medium (UTM) (COPAN, Brescia, Italy). Viral RNA was extracted from 300 µl of UTM with Nimbus automatic system (Arrow Diagnostics, Genoa, Italy), according to the manufacturer’s instructions. Amplification was performed on BioRad CFX PCR machine (Bio-Rad Laboratories S.r.l., Milan, Italy) using the Allplex™ 2019-nCoV Assay (Seegene Inc. Seoul, Korea) reagents which detects conserved regions in ORF1ab, E and N genes of the SARS-CoV-2 genome. Cycle threshold (Ct) values were automatically calculated using the 2019-CoV Viewer analysis software (Seegene).

### Cells

African green monkey kidney Vero E6 cell line was obtained from Istituto Zooprofilattico Sperimentale (Brescia, Italy) and maintained in Dulbecco’s Modified Eagle Medium (DMEM; Gibco, Thermo Fisher Scientific, Waltham, MA, USA) supplemented with 10% fetal bovine serum (FBS; Gibco, ThermoFisher Scientific) at 37 °C in a humidified atmosphere of 5% CO_2_.

### Virus isolation and infection

PCR-positive nasopharyngeal swabs were diluted 1:2 with DMEM supplied with 1% Penicillin–Streptomycin (Merck, Darmstadt, Germany) and 1% amphotericin B (Merck) before adding to Vero E6 cells. After incubation at 37 °C for 1 h, the inoculum was removed, washed with warm phosphate saline buffer (PBS, Gibco, Thermo Fisher Scientific) twice, and replaced with fresh culture medium containing antimicrobials and 2% FBS. Cells were incubated at 37 °C and observed daily by light microscopy for cytopathic effects (CPE). Cell viability was evaluated by trypan blue exclusion. Cell infection was assessed by quantitative real-time RT-PCR (qRT-PCR). Cell subculturing was performed by seeding rescued cells (1:2) in fresh medium. All procedures were carried-out in a biosafety level-3 (BSL-3) laboratory.

### Viral RNA extraction and qRT-PCR

RNA was extracted from clarified cell culture supernatants (16,000 g x 10 min) using QIAamp Viral RNA^®^ Mini Kit (Qiagen, Hilden, Germany) according to the manufacturer’s instructions. RNA was eluted in 30 μl of RNase-free water and stored at –80 °C until use. The qRT-PCR was carried-out following previously described procedures with minor modifications [13]. Briefly, reverse transcription and amplification of the S gene were performed using the one-step QuantiFast Sybr Green RT-PCR mix (Qiagen) as follows: 50 °C for 10 min, 95 °C for 5 min; 95 °C for 10 s, 60 °C for 30 s (40 cycles) (primers: RBD-qF1: 5′-CAATGGTTTAACAGGCACAGG-3′ and RBD-qR1: 5′-CTCAAGTGTCTGTGGATCACG-3). Standard curve was obtained by cloning the receptor binding domain of S gene (primers: RBD-F: 5′-GCTGGATCCCCTAATATTACAAACTTGTGCC-3′; RBD-R: 5′-TGCCTCGAGCTCAAGTGTCTGTGGATCAC-3′) into pGEM T-easy vector (Promega, Madison, WI, USA). A standard curve was generated by determination of copy numbers derived from serial dilutions (10^3^–10^9^ copies) of the plasmid. Each quantification was run in triplicates.

### Immunofluorescence analysis

Infected Vero E6 cells were seeded (5x10^4^ cells/well) in 8-well chamber slides (Becton–Dickinson, Franklin Lakes, New Jersey, USA). Twenty-four h later, cells were fixed with 2% paraformaldehyde in PBS for 10 min, permeabilized with 0.1% Triton X100 in PBS, and saturated with 3% BSA, 0.1% Tween 20 in PBS. For staining, cells were incubated overnight with a human serum containing IgG to SARS-CoV-2 (1:200 dilution) followed by Alexa Fluor 488-conjugated anti-human IgG (Thermo Fisher Scientific). Nuclei were counterstained with 4′,6-diamidino,2-phenylindole (DAPI, Merck). Cells were analyzed using a Leica (Wetzlar, Germany) TCS SP5 laser scanning fluorescence microscope and the imaging software Leica Application Suite.

### Metagenomic analysis

Total RNA was extracted from clarified cell culture supernatants (16,000 g x 10 min) using RNeasy^®^ Mini kit (Qiagen) following manufacturer’s guidelines. RNA has been eluted in 30 μl and stored at –80 °C until use.

Randomly amplified cDNA was generated using Sequence-independent Single-Primer Amplification (SISPA) Round A/B technique as described [14] with minor modifications. Briefly, in Round A, RNA has been retrotranscribed with SuperScript III Reverse Transcriptase (Thermo Fisher Scientific), using 40 pmol of Sol-PrimerA (5′-GTTTCCCACTGGAGGATA-N9-3′). Second-strand DNA synthesis was obtained using Sequenase DNA polymerase (Thermo Fisher Scientific) by incubating first strand cDNA at 37 °C for 8 min in 5 μl of Sequenase Mix #1 (1 μl 5 × Sequenase Buffer, 3.85 μl H_2_O, 0.15 μl Sequenase enzyme). To favour the complete second strand synthesis, 0.6 μl of Sequenase Mix #2 (0.45 μl Sequenase Dilution Buffer, 0.15 μl Sequenase Enzyme) was added to the previous mix and a further incubation at 37 °C for 8 min was performed. In round B reaction, 5 μl of Round A-labeled cDNA were subjected to amplification using AmpliTaq^®^ Gold (Thermo Fisher Scientific) and 100 pmol Sol-PrimerB (5′-GTTTCCCACTGGAGGATA-3′) in a 50 μl final volume. PCR conditions were as follows: 95 °C for 10 min; 94 °C for 30 s, 50 °C for 45 s, and 72 °C for 60 s (40 cycles), 72 °C for 7 min. To maximize the recovery of fragments > 200 bp, PCR products were purified using 1.8 × ratio AMPure XP beads (Agencourt, Beckman Coulter Inc., USA).

Purified products were quantified using the Qubit^®^ DNA HS Assay Kit (Thermo Fisher Scientific), then genomic libraries were prepared using Nextera DNA Flex kit (Illumina, San Diego, CA). Sequencing was performed using an Illumina MiniSeq^®^ platform (Illumina) generating 2x150 bp paired-end reads. Raw data were checked for quality using FastQC (https://www.bioinformatics.babraham.ac.uk/projects/fastqc/) and for bacterial, archaeal, and viral genomes correspondence using Kraken2 with MiniKraken2 Database [15], then were trimmed with Trimmomatic ver. 0.38 for quality (Q score > 25) and length (> 36 bp) by (i) removal of any adaptor sequences; (ii) removal of leading bases with PHRED < 25 and of trailing bases with PHRED < 25; (iii) clipping of the remainder of the read when a sliding window of 20 bases has average PHRED < 25; (iv) removal of reads with length < 36 bases [16]. Paired-end trimmed reads were analyzed with Geneious^®^ software (version 11.1.5) (Biomatters Ltd, New Zealand). Consensus sequence was reconstructed and mapped to the SARS-CoV-2 reference sequence NC_045512.2 using Bowtie2 in sensitive-local mode with consensus threshold at 65% [17].

The variant calling was carried out by the Variant Finder Tool (Geneious) filtering out variants with a *p* value greater than 0, using a minimum variant frequency of 0 and default parameters for Maximum Variant p-value (10^−6^). The minimum sequencing coverage for each variant position was 10 reads. Variant’s frequencies were evaluated as a sum of variant frequencies at that position. Each sample was processed and analyzed in two independent experiments.

### Phylogenetic analysis

Public SARS-CoV-2 complete genome sequences (> 29 Kb), available up to June 2, 2020 were retrieved from the GISAID. Low-quality genomes and nearly identical sequences (genetic similarity > 99.99%) were excluded, obtaining a global dataset of 3,171 public genomes plus the 2 novel genomes reported in this study. Sequences were aligned by MAFFT (FF-NS-2 algorithm) using default parameters [18]. The alignment was manually curated to remove artifacts at the ends and within the alignment using Aliview [19]. Phylogenetic analysis was performed using IQ-TREE (version 1.6.10) under the best fit model according to Bayesian Information Criterion (BIC) indicated by the Model Finder application implemented in IQ-TREE [20]. The statistical robustness of individual nodes was determined using 1000 bootstrap replicates. Lineages assessment was conducted using **P**hylogenetic **A**ssignment of **N**amed **G**lobal **O**utbreak **LIN**eages tool available at https://github.com/hCoV-2019/pangolin [21].

### Statistical analysis

Ct medians were calculated in the early and late epidemic periods for E, RdRP and N gene targets. Statistical analyses were performed using a two tailed unpaired *t*-test. Differences were considered significant at p< 0.05.

Statistics and graphical rendering of plots were performed using Prism 8 software (GraphPad Software, La Jolla, CA, USA).

## Results

### Brescia COVID-19 epidemic

**Fig. 1:**
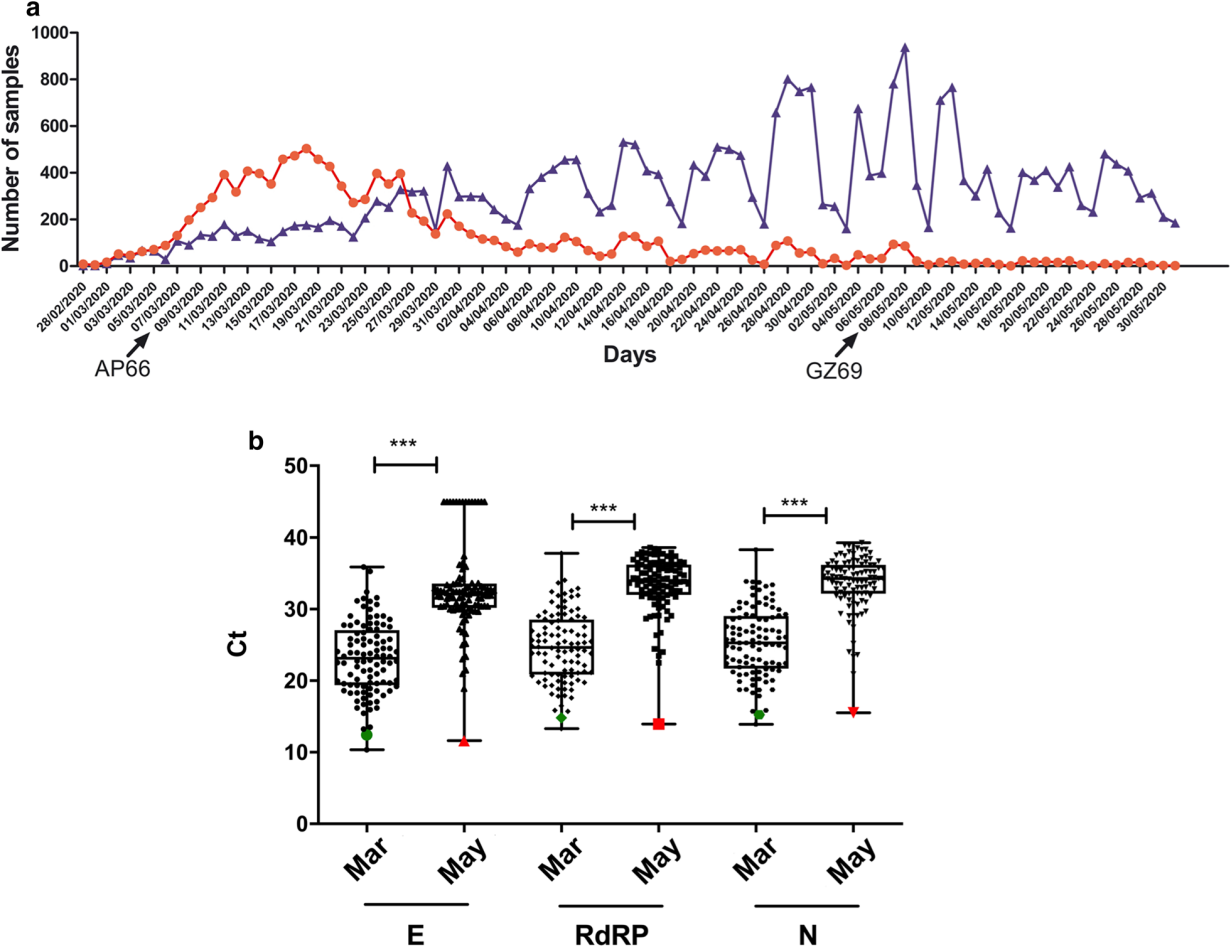
Characterization of SARS-CoV-2 samples analyzed at the Brescia Civic Hospital. Nasopharyngeal swabs were routinely collected and evaluated for the presence of SARS-CoV-2 RNA using a multitarget (E, RdRP, N genes) real time PCR approach as described in the Methods section. **a** Graphs represent the absolute number of specimens that were tested daily. Positive SARS-CoV-2 samples are shown in red; negative SARS-CoV-2 samples are shown in blue. Horizontal axis indicates time in daily interval from February 28 through May 31, 2020. Arrows show the days in which AP66 and GZ69 samples were respectively collected. **b** One-hundred randomly selected SARS-CoV-2 positive samples collected at the early peak of infection epidemic (March 2020) and at the late stage of infection epidemic (May 2020) in Brescia region were stratified according to the Ct values obtained from E, RdRP and N genes. In box and whiskers graphs, boxes extend from the 25^th^ to the 75^th^ percentiles, lines indicate the median values, and whiskers indicate the range of values. (*** p < 0.001). Green signs indicate Ct from AP66 sample; red signs indicate Ct from GZ69 sample

We started to analyze nasopharyngeal swabs for the presence of SARS-CoV-2 genome from the Brescia region on February 28, 2020. From this date to May 31, we have analyzed a total of 40,730 samples and, among them, 11,344 samples (28.8%) were found positive for the presence of SARS-CoV-2 genome. As shown in Fig. 1a, most of the positive samples were found in March (N = 8,398, 62.0% of the 13,547 analyzed), possibly representing the population that acquired the infection before a strict lock-down strategy was applied in Italy. Later on, positive cases were still present but the percentage of the SARS-CoV-2 positive samples among the total analyzed was drastically reduced. In May, only 611 out of 12,705 analyzed specimens (4.8%) were found positive. We also sought to evaluate if the two epidemic phases differed not only for number of positive cases but also, as recently observed in patients’ cohorts in Milan [22], for amounts of detected viral RNA. To this aim, we analyzed Ct values observed in 100 randomly selected positive samples derived from the peak of epidemic (March 2020) and other 100 positive samples detected in May 2020. For each sample, we stratified Ct values observed for the 3 molecular targets detected by the Seegene diagnostic reagent (RdRP, E, N genes). Median Ct values for all the 3 target genes observed in March was significantly lower (E gene median Ct: 23.3, range 10.3-35.9; RdRP gene median Ct: 24.6, range 13.3-37.8; N gene median Ct: 25.3, range 13.9-38.3) than the median Ct values detected in samples collected in May (E gene median Ct: 32.1, range 11.6-45.0; RdRP gene median Ct: 34.0, range 13.9-38.6; N gene median Ct: 34.3, range 15.5-39.3) (Fig. 1b). In the later stage of epidemic one sample (GZ69), was strikingly different from all the other nasopharyngeal swabs analyzed. GZ69 specimen showed a very high amount of virus with Ct values (E gene Ct: 11.6; RdRP gene Ct: 13.9; N gene Ct: 15.5) that fell in the lower quartile of samples analyzed in the early epidemic period. Interestingly, GZ69 subject was completely asymptomatic being his sample obtained during a systematic healthcare workers screening on May 8.

### Persistent infection of SARS-CoV-2 GZ69 isolate in Vero E6 cells

High viral load detected in GZ69 sample allowed us to isolate SARS-CoV-2 on Vero E6 cells. For comparison, we have also isolated a second virus (SARS-CoV-2 AP66), obtained from an age and gender-matched hospitalized patient infected in the early epidemic (March 9, 2020). AP66 sample showed a virus level superimposable to the amount of virus detected in GZ69 sample. AP66 sample Ct values were also comprised in the lower percentile of the early epidemic period (Fig. 1) (E gene Ct: 12.4; RdRP gene Ct: 14.8; N gene Ct: 15.3).

**Fig. 2:**
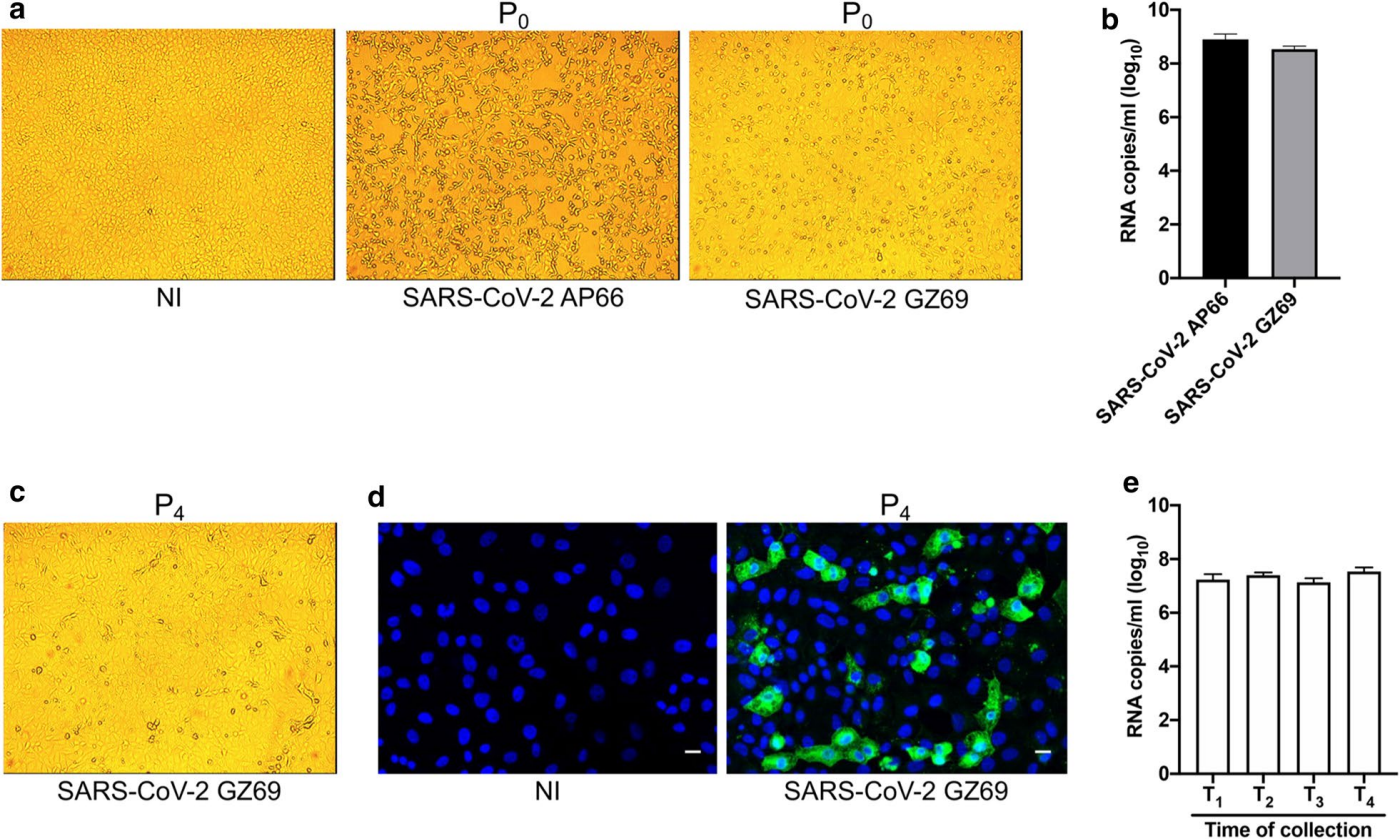
Persistence of SARS-CoV-2 GZ69 in Vero E6 cells. **a** CPE induced by SARS-CoV-2 AP66 and SARS-CoV-2 GZ69 in Vero E6 at P_0_ (72 h p.i.) (Original magnification 10x). **b** SARS-CoV-2 RNA copy number was calculated in cell supernatants collected at P_0_ by qRT-PCR. Values represent the S gene copies/ml mean ± SD of the triplicate. **c** Bright field of persistent infected SARS-CoV-2 GZ69 at P_4_ (Original magnification 10x). **d** Immunofluorescence of Vero E6 cells persistently infected with SARS-CoV-2 GZ69 at P_4_. Images display SARS-CoV-2 signals in green and cell nuclei in blue (Scale Bar 20 µm). **e** SARS-CoV-2 RNA copy number was calculated in cell supernatants collected prior to cell subculture by qRT-PCR. Times of supernatant collection are indicated with a T. Values represent the S gene copies/ml mean ± SD of the triplicate. *NI* not infected

Surprisingly, the SARS-CoV-2 GZ69 isolate showed a much lower aggressiveness as compared to AP66. Indeed, as expected when samples’ SARS-CoV-2 viral load is very high, isolation of AP66 in Vero E6 (P_0_) led to a CPE appearance as early as 48 h post infection (p.i.), quickly spreading to the entire cell monolayer by 72 h p.i. On the contrary, despite comparable amounts of virus were present in AP66 and GZ69 specimens, Vero E6 viability was only slightly altered by GZ69 isolation, being CPE at 72 h p.i. limited to a few elements within a well-preserved cell monolayer (Fig. 2a). Interestingly, at 72 h p.i. (time 0, T_0_), no difference in the rescue of AP66 (5.2 x 10^8^ genome copies/ml of cell culture supernatant) and GZ69 (3.8 x 10^8^ genome copies/ml of cell culture supernatant) viral progenies was observed, thus excluding that lack of CPE in Vero E6 infected by GZ69 was due to an inefficient isolation process or to a slower viral replication kinetic (Fig. 2b). Monitoring of cells up to 8 days p.i. confirmed that infection of Vero E6 cells with SARS-CoV-2 GZ69 did not result in a clear CPE, thus suggesting that a persistent infection has been established. To confirm this hypothesis, cells were regularly split every 4 days up to passage 4 (P_4_) and cellular supernatants were collected prior to each cell subcultivation, from T_1_ to T_4_. GZ69-infected Vero E6 cells rescued at each passage were largely viable (75-85%) and did not exhibit a slower growth in comparison to the not infected counterpart (Fig. 2 c, d). Of note, a continuous release of SARS-CoV-2 GZ69, ranging from 1.4 x 10^7^ to 3.8 x 10^7^ genome copies/ml of cell culture supernatant, was also observed (Fig. 2e). This result shows that the absence of CPE in Vero E6 cells does not reflect the SARS-CoV-2 GZ69 replicative capacity.

### Genetic mutation of SARS-CoV2 GZ69

**Fig. 3:**
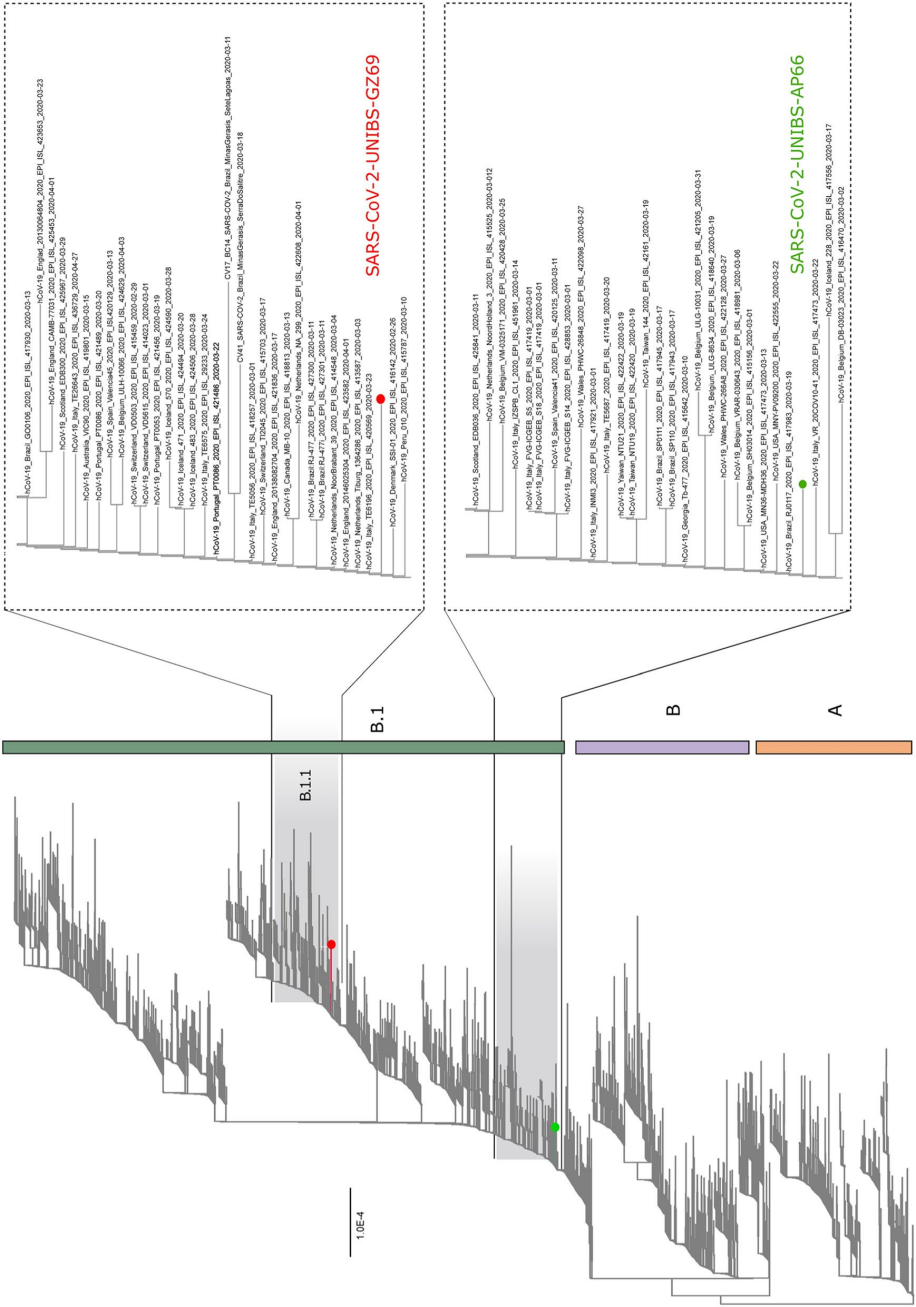
Phylogenetic analysis of SARS-CoV-2 AP66 and SARS-CoV-2 GZ69 isolates. Maximum likelihood tree of 3,173 sequences of SARS-CoV-2 sampled worldwide. The two strains evaluated in this study were marked with the colored circles. On the right side, zooms of the clades containing the two Italian SARS-CoV-2 isolates are shown

A Whole genome sequencing (WGS) approach was applied to evaluate if differences in CPE induced by SARS-CoV-2 GZ69 and SARS-CoV-2 AP66 were mirrored by genetic variations between the two isolates. Metagenomic sequencing of the isolates (T_0_) was performed using a MiniSeq Illumina platform. Raw sequence reads were trimmed for quality (Q > 25) and length (> 36 bp) and aligned to the complete genome of SARS-CoV-2 Wuhan-Hu-1 isolate (Genbank accession number: NC_045512.2) using Bowtie2. Viral genome was covered at 99.9% for both isolates with no significative gaps observed in any coding positions. To analyze the GZ69 and AP66 SARS-CoV-2 genomes in a comprehensive phylogenetic context, we performed a maximum likelihood (ML) analysis on a dataset containing 3,171 sequences deposited in GISAID up to June 2, 2020. Our estimated ML phylogeny identified two major clades, lineages A and B as per the nomenclature recently proposed, branching at the root of the tree [23]. This analysis showed that SARS-CoV-2 AP66 isolate clustered in the B1 clade which includes most of the Italian sequences, together with sequences derived from other European countries and United States. The SARS-CoV-2 GZ69 genome sequence appeared, in contrast, to be located in a different cluster being assigned to SARS-CoV-2 sub-lineage B.1.1. Again, this recent sub-lineage mainly includes genome sequences from Italy, Europe and United States. In the tree, some sequences from other SARS-CoV-2 collected in Lombardy area, segregated in clusters different from those containing the two novel sequences characterized in this study (Fig. 3). This suggests that, in this geographical area, multiple SARS-CoV-2 introductions have occurred through time.

**Fig. 4:**
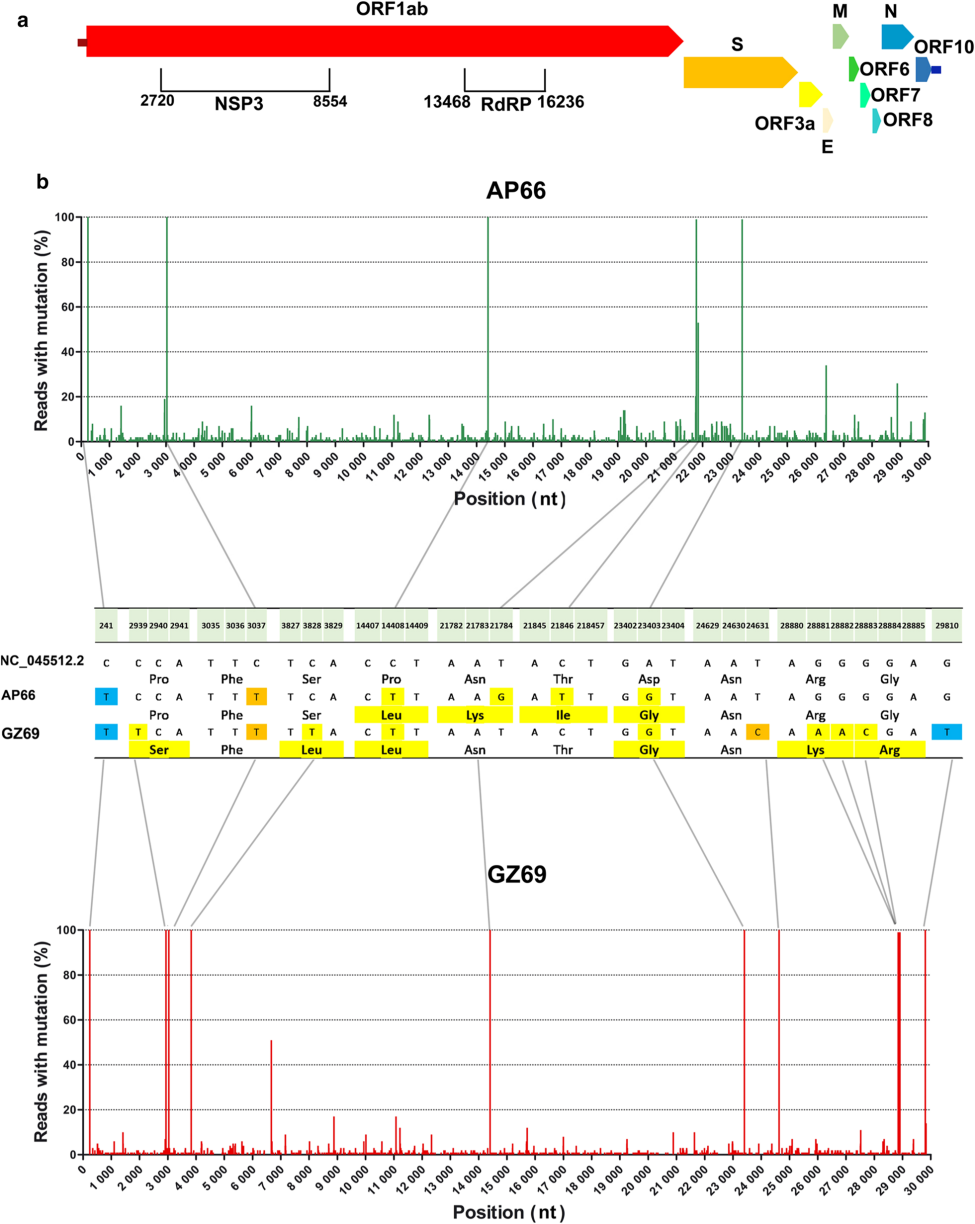
Inspection of genomic variability of SARS-CoV-2 AP66 and SARS-CoV-2 GZ69 isolates. **a** A colour-coded scheme of the SARS-CoV-2 genetic regions, annotated as in the reference genome NC_045512.2. ORF1ab domains where mutations in SARS-CoV-2 AP66 and SARS-CoV-2 GZ69 occurred are indicated with square brackets. Numbers indicate nucleotide (nt) positions. **b** Sequence variability detected in SARS-CoV-2 AP66 (upper green graph) and SARS-CoV-2 GZ69 (lower red graph). Vertical axis represents the  % of reads displaying mutations in comparison to the reference sequence NC_045512.2 at each nt position detected, as assessed by Next Generation Sequencing. Nt positions in the SARS-CoV-2 genome are reported in the horizontal axis. The central table displays nt positions where a substitution in SARS-CoV-2 AP66 and SARS-CoV-2 GZ69 in comparison to NC_045512.2 occurs (Numbers within the light green box). Nt substitutions in non-coding regions are in blue; silent substitutions are in orange; non-synonymous nt substitutions and the correspondent amino acid changes are highlighted in yellow

Alignment analyses [24] showed that, with respect to the Wuhan-Hu-1 Reference Genome NC_045512.2, SARS-CoV-UNIBS-2 AP66 consensus sequence displayed 6 nucleotide substitutions (C241T; C3037T; C14408T; T21784G; C21846T; A23403G) leading to 4 non-synonymous changes (ORF1ab polypeptide: P4715L; S protein: N74K, T95I, D614G). Three out of 6 nucleotide substitutions reside within the gene encoding the S protein and all of them gave origin to non-synonymous amino acid changes. The other non-synonymous substitution observed is located at the ORF1ab coding region, within the RdRP domain. This mutation (P4715L), together with the D614G in the S protein, are recurrent mutations that have emerged in Europe starting from February 2020 as recently clearly described by Pachetti et al. [25]. In agreement with this finding, mutations at nt positions 14,408 and 23,403 were present also in the late (May 2020) SARS-CoV-2-UNIBS-GZ69 viral variant. Less pathogenic GZ69 consensus sequence differed from Wuhan-Hu-1 isolate in 11 nucleotide positions (C241T; C2939T; C3037T; C3828T; C14408T; A23403G; T24631C; G28881A; G28882A; G28883C; G29810T) and 6 of them were non-synonymous (ORF1ab polypeptide: P892S, S1188L, P4715L; S protein D614G; N Protein: R203K, G204R). Interestingly, SARS-CoV2-UNIBS-GZ69 consensus differed from the early SARS-CoV-2-UNIBS-AP66 variant in 9 positions (C2939T; C3828T; G21784T; T21846C; T24631C; G28881A; G28882A; G28883C; G29810T) and these genetic variations have led to 4 non-synonymous substitutions in the ORF1ab (P892S, S1188L), in the S protein (K74N, I95T) and in the N protein (R203K, G204R) (Fig. 4).

To exclude that the mutations observed in the SARS-CoV-2 GZ69 isolate were introduced during viral replication in vitro we have performed WGS directly on GZ69 nasopharyngeal swab. In comparison to Wuhan-Hu-1 isolate, consensus sequence of ex vivo GZ69 nasopharyngeal sample showed the same 11 nucleotide substitutions identified in the SARS-CoV-2 GZ69 isolate. Of note, substitutions identified at each nucleotide position in the GZ69 clinical sample were present in 100% of the reads analyzed, thus excluding that in vitro isolation may have selected a single SARS-CoV-2 intra-host variant.

## Discussion

Viral mutation rates/nucleotide substitutions/cell infection ranges for RNA viruses approximately between 10^−6^ and 10^−4^ [26]. The exception to this rule is provided by coronaviruses, encoding a complex RNA-dependent RNA polymerase that has a 3′ exonuclease domain [26]. Thousands of SARS-CoV-2 whole genomes have been sequenced up to date proving crucial for tracing viral origin and evolution. Surprisingly, the SARS-CoV-2 global population seems to have accumulated only moderate genetic diversity at this stage of the COVID-19 pandemic, possibly reflecting a relative recent common ancestor [27]. An estimated mutation rate underlying the global diversity of SARS-CoV-2 of approximately 6 x 10^−4^ nucleotides/genome/year has been recently calculated [27]. This is largely unremarkable for an RNA virus [26, 28], even if we consider that SARS-CoV-2 has the capability of proofreading the errors of RNA polymerase by the activity of the multidomain non-structural protein (nsp)14 [29, 30]. Nevertheless, the examination of a dataset of 7,666 public genome assemblies identified regions of accumulating diversity, with 198 recurrent mutations emerged independently, worldwide, multiple times (homoplasies). Of these, nearly 80% of the recurrent mutations produced non-synonymous changes at the protein level, suggesting possible ongoing adaptation of SARS-CoV-2 to the human host [27]. In a recent study, metatranscriptome sequencing on bronchoalveolar lavage fluid samples of 8 patients showed that the number of intra-host variants ranged from 0 to 51, with a median of 4, suggesting a high evolution rate of the virus [31]. This is not surprising as when the level of infection of a population becomes significant, some individuals become multiply infected with different variants of the virus [32]. Moreover, analysis of viral isolates highlighted the presence of quasispecies showing novel genetic mutations despite the relatively early sampling dates [11, 12] thus indicating that the true diversity of the viral strains is largely underappreciated [11].

The wide variety of clinical symptoms characterizing COVID-19 patients makes extremely difficult to establish a genotype-to-phenotype link. This knowledge is crucial for understanding infectious mechanisms used by SARS-CoV-2 and directing the strategy for drug and vaccine development. Therefore, studying the mutational impact of viral isolates in vitro becomes fundamental to this aim. The high viral load detected in nasopharyngeal swab obtained lately in the Italian epidemic has allowed, for the first time to our knowledge, to isolate SARS-CoV-2 from an asymptomatic subject and to conduct in vitro experiments to study its replicative features. Experiments were performed on Vero E6 cells since they are fully susceptible to SARS-CoV-2 infection and provide a valuable substrate to avoid as much as possible constraints to the virus due to the absence of the interferon response [33] to which SARS-CoV-2 is highly sensitive [34, 35]. Surprisingly, the novel SARS-CoV-2 GZ69 isolate did not induce any cytopathic effect on Vero E6 cells despite a high viral load in the culture supernatant. The viral load continued to be sustained, at levels usually reached by cytopathic viruses, even when cells were passaged several times. This finding attests for the unprecedented ability of the novel isolate to manipulate cell machinery to circumvent cell death and demands for further studies to understand the molecular mechanisms underlying its persistent infection. Nidovirales and, among them, coronaviruses are prone to establish persistence both in vivo and in vitro [36]. It is well admitted that persistence results from adaptations of both the host cell and the virus [37] and that mutations in the viral genome concur to the persistence [38]. This has been clearly demonstrated also for SARS-CoV, the phylogenetically closest virus to SARS-CoV-2, where a point mutation is stabilized during the establishment of persistence [39, 40], suggesting that even a single but crucial amino acid change may be advantageous for virus adaptation. In this paper, we have highlighted that SARS-CoV-2 GZ69 and cytopathic SARS-CoV-2 AP66 display some amino acid differences. These variations allowed us to categorize SARS-CoV-2 GZ69 and SARS-CoV-2 AP66 in different phylogenetic subgroups and suggest that SARS-CoV-2 GZ69 may have evolved, during time, from the B1 genetic group where SARS-CoV-2 AP66 and many other Italian strains are classified.

Mutations in SARS-CoV-2 S gene have been correlated with its attenuation or enhanced aggressiveness [12]. In agreement with the evidence that SARS-CoV-2 GZ69 isolate showed no relevant impairment in infectivity, we observed that it maintained a “wild type” S sequence; the fixed “european” D614G substitution was, in fact, the only difference detected with respect to Wuhan reference strain. Comparison between the SARS-CoV-2 GZ69 and SARS-CoV-2 AP66 sequences revealed the presence of 4 more non-synonymous substitutions. Mutations at amino acid residues 892 (P to S) and 1188 (S to L) have not yet been described worldwide. They both reside within the large nsp3, whose mutational events have been considered a potential mechanism differentiating COVID-19 from SARS [41]. In particular, mutation S1188L is comprised within the SARS-CoV-2 macrodomain (Mac1, residues 1023-1197 of polyprotein 1a), a domain that is present in all coronaviruses. Mac1 binds and removes ADP-ribose from post-translationally modified cellular proteins and this activity counteracts host antiviral ADP-ribosylation [42]. Remarkably, Mac1 mutation in SARS-CoV does not interfere with virus replication in Vero E6 cell cultures [43] but mutant virus is highly attenuated in vivo [44–46]. These results attest that SARS-CoV Mac1 is largely dispensable for viral replication but is required for the pathogenesis and likely promotes virulence by countering the mammalian innate immune response. It is therefore tempting to speculate that the mutation observed in Mac1 of SARS-CoV-2 GZ69 may take part in different CPE induced on Vero E6 described herein.

The other differences between SARS-CoV-2 AP66 and SARS-CoV-2 GZ69 reside within the N gene, where 2 consecutive amino acids (203-204) were replaced. These substitutions are located in the N2a linker domain which is uniquely tolerant of mutations, in keeping with its likely structural role as a disordered linker between the RNA-binding N1b domain and the N2b dimerization domain [47]. In agreement with this, mutations observed in the N gene are not unique of GZ69 viral strain being already detected in other SARS-CoV-2 isolates worldwide [48]. However, no relationship between the presence of these substitutions and viral pathogenicity has yet been assessed. In this context, it is worth to note that, in SARS-CoV, these residues are part of putative phosphorylation sites and that this epitope is involved in interaction with different cellular enzymes such as cyclin-dependent kinase (CDK) and glycogen synthase kinase-3 (GSK3) [49]. Furthermore, mutational analyses involving residue 204 have provided substantial evidence that the N protein of the SARS-CoV binds and inhibits the activity of the cyclin-CDK complex, resulting in the down-regulation of the S phase gene products and the subsequent inhibition of S phase progression in human cells [50]. The mutational events occurred in SARS-CoV-2 GZ69 at 203-204 residues may reduce the anti-proliferative properties of N protein, favouring cell survival and, eventually, viral persistence. The recent availability of an infectious cDNA clone of SARS-CoV-2 [51] makes possible reverse genetics attempts to determine whether replacement of the above described amino acid residues within a well characterized SARS-CoV-2 molecular clone may drastically alter the viral aggressiveness.

## Conclusions

In this paper, we have shown the existence of a SARS-CoV-2 variant, isolated in the late Italian epidemic from an asymptomatic health care worker, capable of persistent replication in Vero E6 cells in the absence of CPE. SARS-CoV-2 GZ69 variant displays several point mutations that may account for its unique features.

The identification of the peculiar SARS-CoV-2 GZ69 strain in the late epidemic highlights the need to better characterize viral variants circulating among asymptomatic or paucisymptomatic individuals. This will allow to identify critical genetic mutations that might be a part of the viral adaptation process eventually leading to changes in virus pathogenicity. This distinct outcome could be used to search for host gene expression and/or signaling pathways playing a role in the establishment of viral persistence. Taken together, these results could pave the way to future studies aimed at analyzing the selection process favouring viral mutations in the human host.

## Data Availability

Novel genome sequences described in this manuscript are available at GenBank (SARS-CoV-2-UNIBS-AP66: ERR4145453; SARS-CoV-2-UNIBS-GZ69: ERR4298965; SARS-CoV-2-UNIBS-GZ69NPS: ERR4472641).

## Abbreviations

SARS-CoV-2: Severe acute respiratory syndrome coronavirus 2
COVID-19: Coronavirus disease 2019
WGS: Whole genome sequencing
UTM: Universal transport medium
Ct: Cycle threshold
PBS: Phosphate saline buffer
CPE: Cytopathic effect
BSL-3: Biosafety level-3
qRT-PCR: Quantitative real-time Reverse Transcriptase-PCR
DAPI: 4′,6-diamidino,2-phenylindole
SISPA: Sequence-Independent Single Primer Amplification
BIC: Bayesan information criterion
T: Time
P: Passage
p.i.: Post infection
nsp: Non-structural protein
CDK: Cyclin-dependent kinase
GSK3: Glycogen synthase kinase-3
nt: Nucleotide
NI: Not infected
